# Altered expression pattern of miR-29a, miR-29b and the target genes in myeloid leukemia

**DOI:** 10.1186/2162-3619-3-17

**Published:** 2014-07-01

**Authors:** Ling Xu, Yan Xu, Zhenyi Jing, Xu Wang, Xianfeng Zha, Chengwu Zeng, Shaohua Chen, Lijian Yang, Gengxin Luo, Bo Li, Yangqiu Li

**Affiliations:** 1 Institute of Hematology, Medical College, Jinan University, Guangzhou 510632, China; 2 Key Laboratory for Regenerative Medicine of Ministry of Education, Jinan University, Guangzhou 510632, China; 3 Department of clinical laboratory, First Affiliated Hospital, Jinan University, Guangzhou 510632, China

## Abstract

**Objectives:**

The miR-29 family have been demonstrated acting as vital tumor suppressor in multiple cancers as well as regulators in the adaptive immune system. Little is known about their role in leukemogenesis. The purpose of this study is to analyze the expression pattern of miR-29a/29b and its target genes Mcl-1 (myeloid cell leukemia sequence 1) and B-cell lymphoma 2 (Bcl-2) in myeloid leukemia.

**Methods:**

Quantitative real-time PCR was used for detecting genes expression level in peripheral blood mononuclear cells (PBMCs) from 10 cases with newly diagnosed, untreated acute myeloid leukemia (AML) and 14 cases with newly diagnosed, untreated chronic myeloid leukemia (CML) in chronic phase, and 14 healthy individual (HI) served as controls. Correlation between the relative expression levels of different genes have been analyzed.

**Results:**

Significant lower expression of miR-29a/29b and higher expression level of two potential target genes Bcl-2 and Mcl-1 were found in PBMCs from AML and CML patients compared with HI group. In addtion, miR-29a expression in AML was significantly lower than that in CML. Moreover, negative correlation between miR-29a/29b and its target genes have been found. While, positive correlation between relative expression level of miR-29a and miR-29b or Bcl-2 and Mcl-1 were presented in the total 38 research objects.

**Conclusion:**

Down-regulated miR-29a and miR-29b, and accompanying up-regulated Bcl-2 and Mcl-1 are the common feature in myeloid leukemias. These data further support the role for miR-29a/29b dysregulation in myeloid leukemogenesis and the therapeutic promise of regulating miR-29a/29b expression for myeloid leukemia in the future.

## Background

Myeloid leukemia include acute myeloid leukemia (AML) and chronic myeloid leukemia (CML). AML is a heterogeneous disease, characterized by the uncontrolled proliferation of granulocytic, monocytic, megakaryocytic, or rarely, erythroid blast cells [1]. Many different cytogenetic and molecular abnormalities have been demonstrated to be involved in tumorigenesis and tumor progression of this malignancy. The distinct feature of leukemogenesis in AML is differentiation arrest and proliferative advantage of myeloid progenitors [2]. While, CML is a disease of hematopoietic stem cells, arising from a translocation t(9;22)(q34;q11), known as the Philadelphia chromosome. This translocation leads to a juxtaposition of the ABL gene from chromosome 9 and the BCR gene from chromosome 22, resulting in a BCR-ABL fusion gene that codes for BCR-ABL transcripts and fusion proteins with unusual tyrosine-kinase activity. The molecular pathogenesis of CML is well understood, but the molecular mechanism that leads to the gene translocation is unknown [3].

In recent years, microRNAs (miRNAs) have received wide attention as important regulators of gene expression in leukemogenesis and as an anticancer therapy target. miRNAs are 18 to 24 nucleotides (nt) in length that regulate gene expression, for the most part, by targeting mRNAs according to the degree of complementarity with their 3-untranslated region (3’-UTR). miRNAs are involved in the regulation of critical biologic processes, including normal cell homeostasis, cell metastasis and disease pathogensis and progression [4-7]. Recently, expression of miRNAs has been demonstrated to be altered in AML and CML, which may be related to leukemogenesis [8,9]. Distinctive patterns of increased expression and/or silencing of multiple miRNAs are associated with specific cytogenetic and molecular subsets of myeloid leukemia [10]. Changes in the expression of several miRNAs altered in AML have functional relevance in leukemogenesis, with some miRNAs acting as oncogenes and the others as tumor suppressors [11-14]. miR-29 is a very important miRNA family whose members are increasingly recognized as tumor suppressors in a variety of malignancies such as in chronic lymphocytic leukemia (CLL), mantle cell lymphoma (MCL), lung cancer, hepatocelluar carcinoma (HCC) and so on. In contrast to the majority of studies highlighting tumor-suppressive properties [15-18], opposing expression patterns and roles seem to exist in breast cancer and primary melanoma [19,20]. Moreover, it is crucial to identify target genes of miR-29s in different cancer as well as in leukemia in order to decipher cancer-associated cellular pathways and networks that might be regulated by miR-29. Based on those reasons, the number of confirmed targets for miR-29 family members is constantly rising, including many different protein classes ranging from transcription factors, viral proteins to growth factors, structural cell components, anti-apoptosis genes and others [21].

To investigate the encoding of miRNA genes and the targets of miRNA, a lots of computational methods have been devised [22]. Mcl-1 (myeloid cell leukemia sequence 1) and Bcl-2 (B-cell CLL/lymphoma 2) have been predicted as potential target genes of miR-29 family and both of them belong to Bcl-2 family which play central roles in cell death regulation and alterations in their expression and function contribute to the pathogenesis and progression of human cancers [23]. In addition, Mcl-1 have been experimentally validated to be direct miR-29 target in multiple cell types [4,18,24], for example, miR-29a and -29b target Mcl-1 and induces apoptosis in AML have been validated in a research target primary AML samples [24]. Bcl-2, another anti-apoptotic gene of Bcl-2 family, have been up-regulated by the decreased expression level of miR-29 and characterized as target of the miR-29 family in the signaling pathway of hepatocarcinogenesis [18]. It would be interesting to investigate whether Bcl-2 also is one of the target of miR-29 family and contribute to the molecular etiology of AML and CML alone with Mcl-1. Characterization the regulation mechanism between miR-29 and these two anti-apoptosis genes in AML and CML would be important for us to further understand the pathogenetic mechanism in AML and CML.

## Materials and methods

### Samples

The study included 24 de novo and untreated myeloid leukemia patients (10 cases with AML and 14 cases with CML, 11 males and 13 females with a median age of 31 years and a range of 15–71 years). A total of 14 healthy individuals (seven males and seven females with a median age of 30 years and a range of 23–65years) served as the control group. Informed consent was obtained from all patients. The peripheral bloods samples were collected and peripheral blood mononuclear cells (PBMCs) were isolated using Ficoll–Hypaque gradient centrifugation method [25]. RNA was extracted using the Trizol kit (Invitrogen, Carlsbad, CA, USA) and then reverse-transcribed into cDNA for miRNA29 assay using the miScriptIIRT Kit (Qiagen, Duesseldorf, Germany) and reverse-transcribed into first-strand cDNA for target genes assay using random hexamer primers and the high Capacity cDNA Reverse Transcription kit (ABI, Carlsbad,CA, USA) according to the manufacturer’s instructions.

Our research obtained approval by Ethics Committee of Medical School of JINAN University of Guangdong Province of China and all the procedures were conducted according to the guidelines of the Medical Ethics committees of the health bureau of Guangdong Province of China.

### Quantitative real-time quantitative reverse transcription–polymerase chain reaction(qRT–PCR)

SYBR green qRT-PCR assay was used for miR-29a and miR-29b quantification. qRT -PCR assays were carried out in a CFX96 Fast real-time PCR system (Bio-Rad, California, USA) using miScript SYBR Green PCR kit (Qiagen, Duesseldorf, Germany.). Each reaction was performed in a final volume of 25 μl containing 2 μl of the cDNA, 2.5 μl of each primer, 2.5 μl of universal primer and 12.5 μl 2× QuantiTect SYBR Green PCR Master Mix (Qiagen, Duesseldorf, Germany). The amplification profile was denaturation at 95°C for 15 min, followed by 40 cycles of 94°C for 15 s, 55°C for 30s, and 70°C for 30s. At the end of the PCR cycles, melting curve analyses were performed as well as electrophoresis of the products on 1.5% agarose gels in order to validate the specific generation of the expected PCR product. The expression levels of miRNAs were normalized to RNU6B snRNA. Primers used for miRNAs analysis were purchased from Qiagene company and listed in Table 1. The expression levels of Bcl-2, Mcl-1 and the β2-microglobulin (β2-MG) reference gene were determined by SYBR Green I real-time PCR as our previous description [26]. The primers used for real-time PCR for the three gene amplifications were listed in Table 2 and were synthesized by Shanghai Biological Engineering Technology Services Co. Ltd. Random PCR products from all detected genes were also sent to Shanghai Invitrogen Biotechnology Co. for DNA sequence analysis.

**Table 1:** Sequences of miRNA primers used in real-time PCR

***Primer***	***Product imformation***	***Mature miRNA Sequence (5’-3’)***
miR-29a	Hs_miR-29a_1 miScript Primer Assay	UAGCACCAUCUGAAAUCGGUUA
miR-29b	Hs_miR-29b_1 miScript Primer Assay	UAGCACCAUUUGAAAUCAGUGU
RNU6-2	Hs_RNU6-2_11 miScript Primer Assay	ACGCAAATTCGTGAAGCGTT

**Table 2:** Sequences of target gene primers used in real-time PCR

***Primer***	***Sequence***	***PCR products (bp)***
Bcl-2-f	5’-GATGACTTCTCTCGGCGCTACC-3’	126
Bcl-2-r	5’-GTTCACCCCGTCCCTGAAGA-3’	
Mcl-1-f	5’-GGGCAGGATTGTGACTCTCATT-3’	79
Mcl-1-r	5’-GATGCAGCTTTCTTGGTTTATGG-3’	
β2M-f	5’-TACACTGAATTCACCCCCAC-3’	144
β2M-r	5’-CATCCAATCCAAATGCGGCA-3’	

### Statistical analysis

Univariate analyses were done using the student’s t-test to compare means of miR-29a/b expression levels between HI group and AML or CML group,and between two kinds of myeloid leukemia group. Kruskal-Wallis Test was used to analyses the expression levels of Bcl-2 and Mcl-1 among HI, AML and CML group. Pearson correlation and linear regression analysis was used to estimate the correlation between expression level of miR-29a/b and its target genes from all the groups. All statistical tests were two-sided, and *P* values less than 0.05 were considered as statistically significant. All analyses were performed using SPSS software (version 13.0, SPSS).

## Results

### Decreased expression of miR-29a and miR-29b in myeloid leukemia

**Figure 1:**
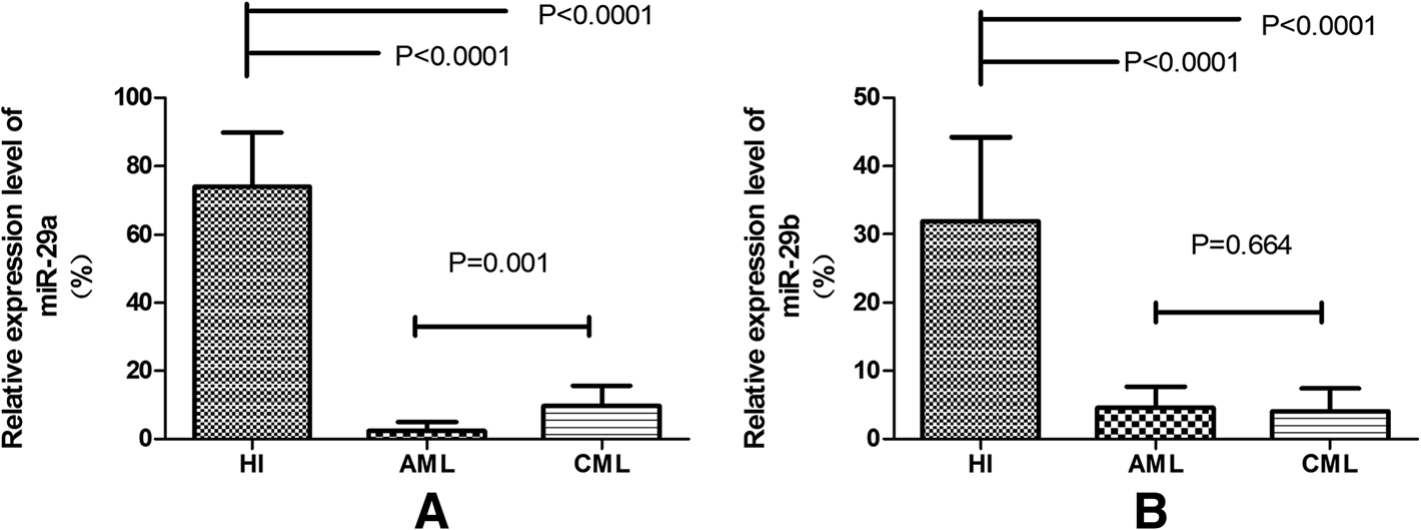
**The relative expression level of miR-29a and miR-29b in PBMCs from AML,CML and healthy (HI) groups. (A)** miR-29a expression level down-regulated in AML and CML; **(B)** miR-29b expression level down-regulated in AML and CML.

Positive PCR products for each detedcted gene from qRT–PCR assay were confirmed by agarose gel electrophoresis, the PCR product direct sequencing also confirmed the specific amplification (data not shown). The results demonstrated the downregulation of miR-29a and miR-29b in myeloid leukemia. miR-29a expression was significantly reduced in PBMCs of AML (2.62 ± 2.63) (p < 0.0001) and CML (9.88 ± 5.73) (p < 0.0001) compared with healthy individuals (HI) (73.97 ± 15.82) (Figure 1A). Similar results were found in miR-29b, its expression levels were significantly lower in AML(4.68 ± 2.94) (p < 0.0001) and CML (4.07 ± 3.28) (p < 0.0001) than that in HI group (31.94 ± 12.25) (Figure 1B). In addtion, miR-29a expression in AML samples was significantly lower than that in CML samples (p < 0.0001). Although miR-29b expression in CML samples also lower than in AML samples, the difference is statistically insignificant (p = 0.664).

### Increased expression of BCL-2 and MCL-1 in myeloid leukemia

**Figure 2:**
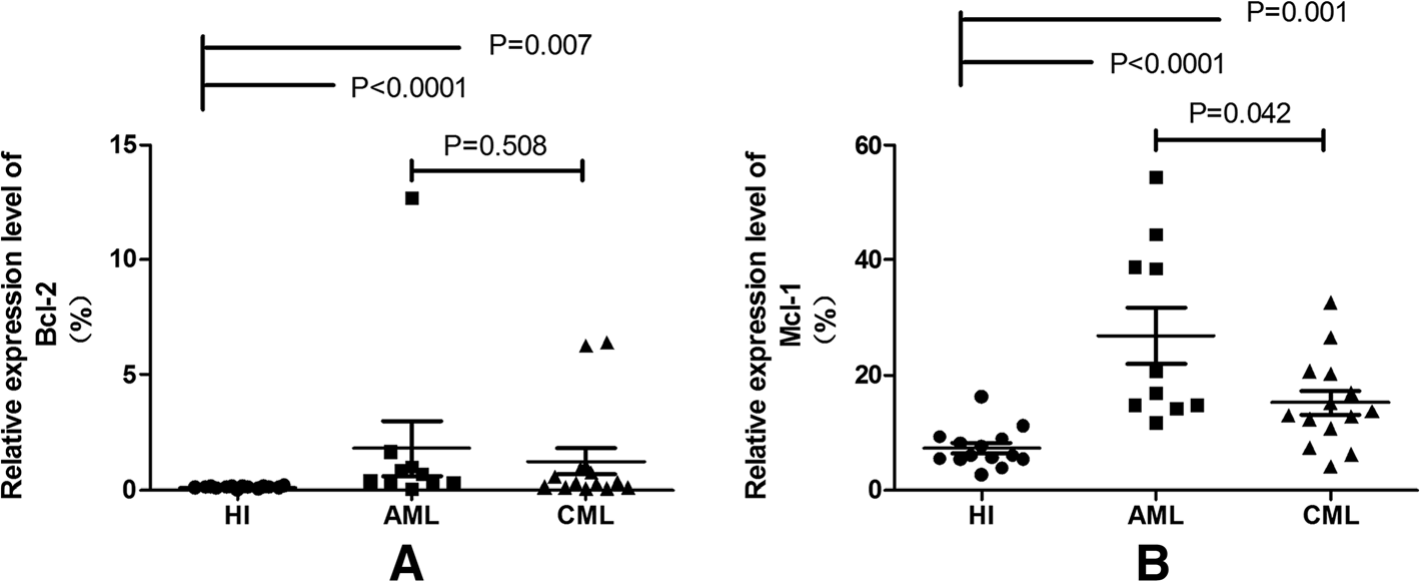
**The relative expression level of Bcl-2 and Mcl-1 in PBMCs from AML,CML and healthy (HI) groups. (A)** Bcl-2 expression level is up-regulated in AML and CML; **(B)** Mcl-1 expression level is up-regulated in AML and CML.

According to the information by miRWalk (http://mirwalk.uni-hd.de/), a comprehensive database that provides predicted as well as validated miRNA binding site information on miRNAs for human, mouse and rat [27]. There are a lot of target genes for miR-29a and miR-29b, such as DNMTs (DNA-methytransferases), T-bet (T-box transcription factor) and CDK6(Cyclin-dependent kinase 6) and so on [16,28,29], among them, we focused on two target genes (Bcl-2 and Mcl-1), both of them are the predicted and validated target genes of miR-29a and miR-29b, and may relate to meyloid leukemogenesis [4,18,24]. We detected the expression levels of Bcl-2 and Mcl-1 gene in PBMCs from AML and CML, as well as HI groups, the data was presented using median. We observed an different expression pattern of the two genes among different groups:Bcl-2 (median: 0.53 > 0.33 > 0.11, p = 0.002) (Figure 2A), Mcl-1 (median: 18.82 > 13.31 > 5.91, p < 0.0001) (Figure 2B). The expression level of Bcl-2 was significant increased in AML (median: 0.53) (p < 0.0001) and CML (median: 0.33) (p = 0.007) groups compared with HI group (median: 0.11). Same results was found in Mcl-1, its expression level was also significant increased in AML (median: 18.82) (p < 0.0001) and CML (median: 13.31) (p < 0.0001) groups in comparison with HI group (median: 5.91). In addition, we can observe a higher expression of Bcl-2 (p = 0.508) and Mcl-1 (p = 0.042) in AML than CML, however, the difference of Bcl-2 have no significant.

### Negative correlation expression of miR-29 and the target genes

**Figure 3:**
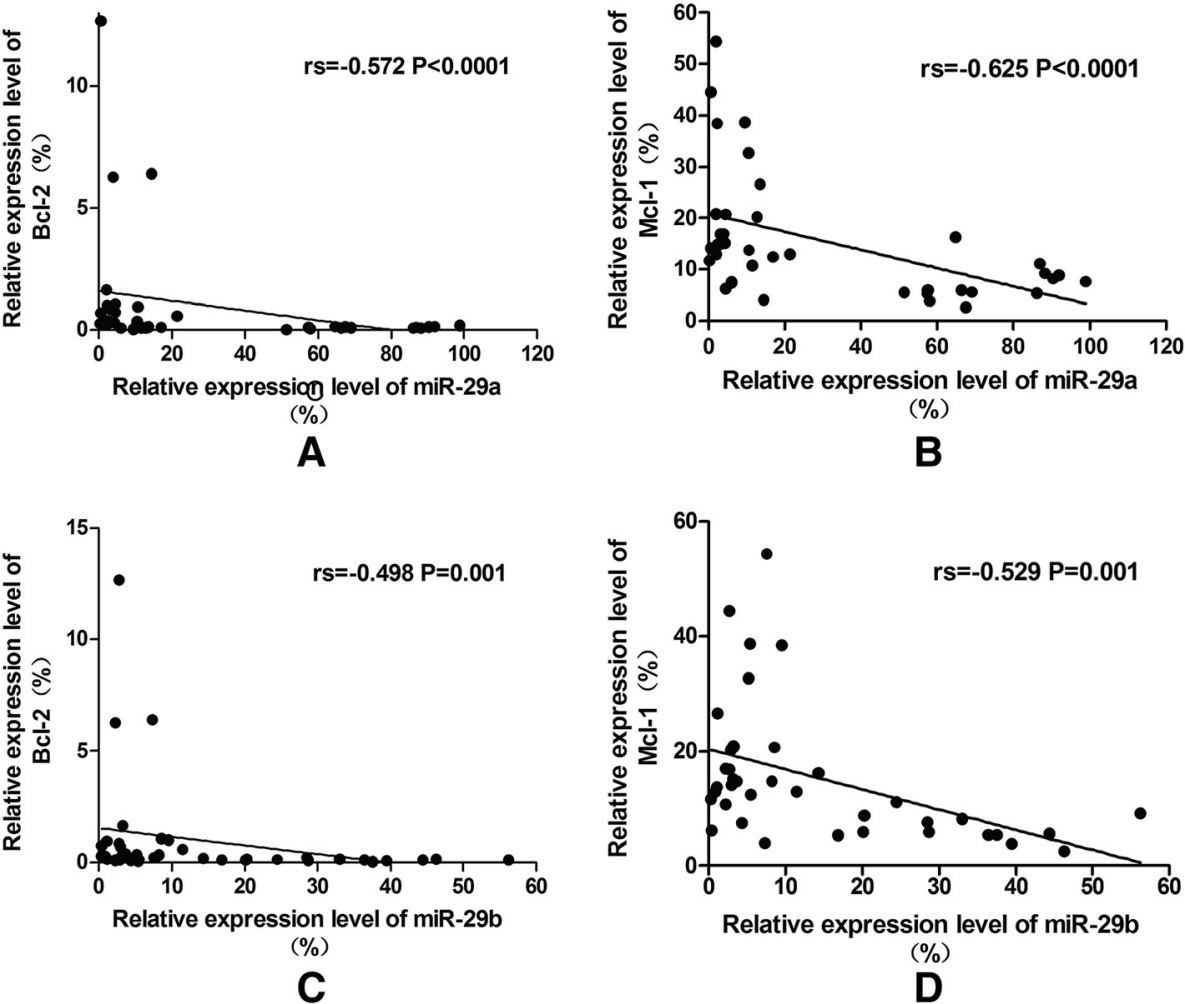
**The negative correlation between miR-29a/29b and target genes in 38 research objects was indicated. (A)** miR-29a vs. Bcl-2; **(B)** miR-29a vs. Bcl-2; **(C)** miR-29b vs. Bcl-2; **(D)** miR-29b vs. Mcl-1.

**Figure 4:**
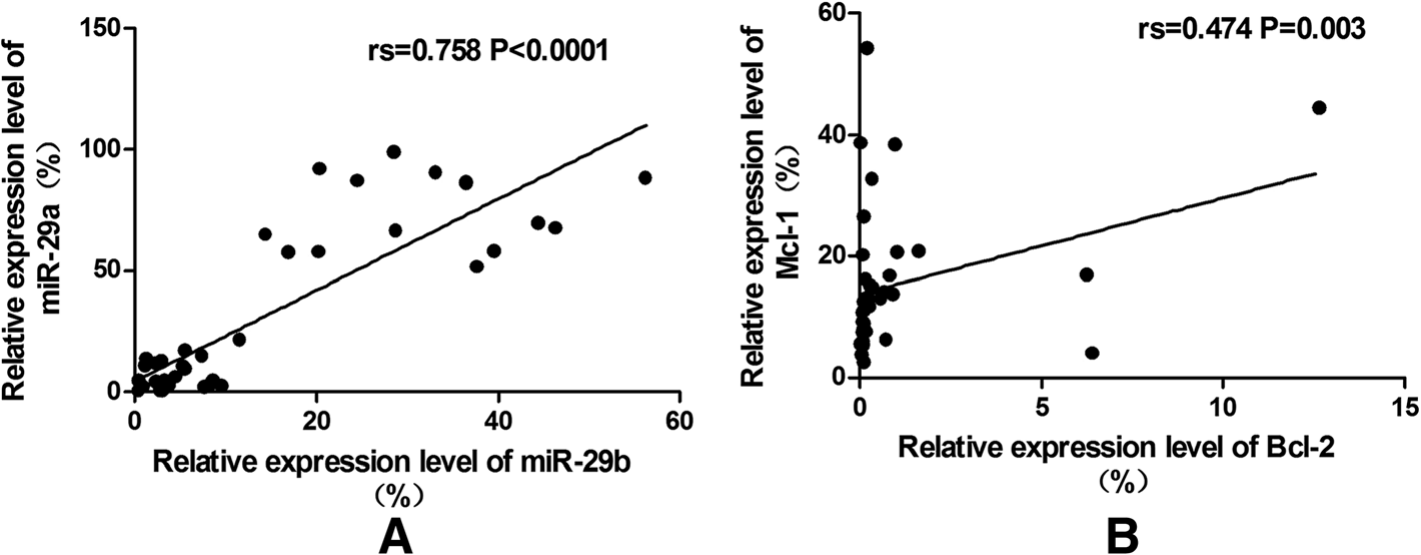
**The positive correlation between miR-29 members as well as between target genes. (A)** A positive correlation between miR-29a and miR-29b was indicated; **(B)** A weak positive correlation between Bcl-2 and Mcl-1 was indicated.

Correlation analysis of the relative expression levels of miR-29a/b and the target genes in the total 38 study objects from HI, AML and CML groups was performed using Spearman’s rank correlation analysis,. A negative expression correlation level for miR-29a and the two target genes (Bcl-2: rs = −0.572, p < 0.0001; Mcl-1: rs = −0.625, p < 0.0001) (Figure 3A and 3B), as well as a negative expression correlation level for genes miR-29b and the two target genes (Bcl-2: rs = −0.498,P = 0.001; Mcl-1: rs = −0.529,p = 0.001) (Figure 3C and 3D) was found, when the correlation analysis was performed in all 38 samples, while there was no significant correlation between the expression levels of miR-29a/b and the two target genes when analysis in the each single group. Moreover, correlation analysis of the relative expression levels of miR-29a and miR-29b in the 38 total research objects showed a positive correlation (rs = 0.758, p < 0.0001) (Figure 4A), however, there was no significant correlation between the expression levels of miR-29a and miR-29b when analysis in the single group. And the expression levels between Bcl-2 and Mcl-1 genes showed an weak positive correlation (rs = 0.474, p = 0.003) (Figure 4B).

## Discussion

Recent studies corroborate on the potential use of miR-29 family as predictive biomarkers for early diagnosis of malignancies and other diseases, however, the exprssion pattern of miR-29 in myeloid leukemia haven’t been assessed accurately and their regulation mechanism in the pathological process of AML remain unclear. In this study, we reported the down-regulation expression of miR-29a and miR-29b in PBMCs of AML patients which is similar to Zhang’s study, they identified miR-29a and miR-142-3p down-regulated in PBMCs from AML, and suggested that may serve as potential biomarkers and therapeutic targets for AML [30]. In addition, several studies showed that the universal down-regulated miR-29 family members have been found in different AML. For example, down-regulation of miR-29a, miR-29b and miR-29c in patients with balanced 11q23 translocations versus all other AML patients have been reported [31], while miR-29a and miR-29c were upregulated in AML with a good prognosis, such as NPMc^+^AML (aberrant cytoplasmic NPM1 localization) compared with NPMc^−^ (unmutated) AML which is with a bad prognosis [32]. All of these results supported that miR-29 family serves as tumor suppressor role in pathogenesis of AML and increasing endogenous expression or ectopic implantation of miR-29a may be a potential strategy for AML treatment. In contrast, another research demonstrated that miR-29a expression in the purified leukemia stem cell (LSC) and non-LSC blast populations were significantly increased compared with common myeloid progenitors (CMP), their data suggest that miR-29a may play a oncogene in human myeloid leukemogenesis [33]. This difference may due to the different control groups used in respective research, or may indicate that miR-29a has different regulating function in different leukemia cells, similar reports such as miR17-92 cluster plays oncogene in B-cell lymphoma, lung cancers, rhabdomyosarcoma and liposarcoma while plays tumor suppressor in hepatocellular carcinomas (HCC) and cervical cancer cell line, further research should be pay attention to appropriate controls and more accurate cell differenciation stage so that we can conduct a more comprehensive analysis for the expression pattern and function of miR-29 in myeloid leukemia.

Few studies described that the expression pattern of miR-29 family members in CML patients [34-37]. Recently, San José-Enériz E et al. reported that miR-29a, miR-29c and other 17 miRNAs were downregulated and could possiblely predict the clinical resistance to imatinib in patients with newly diagnosed CML [34]. In the present study, we also found the down-regulation of miR-29a/miR-29b in PBMCs from CML patients, which is consistent with the microarray results of miR-29b downregulated in leukemia cells form bone marrow of CML blast crisis and RT-PCR results from 5 CML patients reported by Li et al. [36]. From current research data, a prominent downregulation of miR-29a/b in CML might indicate miR-29a/29b as potential tumor suppressor in CML, however, it is needed further comfirmation.

The different expression pattern of miR-29a/miR-29b in AML and CML also have been observed in our study, lower expression of miR-29a in AML compared with CML, similar tendency was found in miR-29b, this may be due to the various stage of maturation of leukemia cells, however, further investigation to characterize the expression feature in different myeloid leukimia subtype is needed with larger sample size.

For further understand the characteristic of expression and regulation of miR-29 in myeloid leukemia, we searched from miRWalk datebase and found two anti-apoptotic genes Mcl-1 and Bcl-2, which are target genes by both miR-29a and miR-29b and highly correlate to myeloid leukemia. As expected we found that Mcl-1 and Bcl-2 were up-regulated in PBMCs from AML patients and negatively correlated with miR-29a/miR-29b expression. Overexpression of Mcl-1 has been reported in cancer, including in AML at relapse [38]. Mcl-1 was negatively regulated by miR-29 s at mRNA level as well as protein level in several different diseases according to 3’-UTR binding [18,24,39]. The current result also showed the same tendency in AML patients at mRNA level, meanwhile, we also showed the same potential regulation pathway in CML which may haven’t been characterized before.

Several previous studies found that high expression of Bcl-2 associate with a low complete remission rate after intensive chemotherapy and with a significantly shorter survival of AML patients [40,41]. The reason of Bcl-2 overexpression may due to chromosomal translocations, gene amplification, epigenetic regulation and so on in lymphoma [42-44]. Besides, loss of endogenous miRNAs repress Bcl-2 gene expression had been documented in CLL and human gastric cancer [45,46]. Recently, a research demonstrated that down-regulation of miR-29 was a frequent event in HCC tissues and independent prognosis predictor for HCC patients, and Bcl-2 and Mcl-1 as functional targets of miR-29 involved in the mitochondrial pathway in miR-29 promoted apoptosis [18]. In this study, we observed a negative correlation between miR-29a/miR-29b and Bcl-2 or Mcl-1 in AML and CML, suggesting that there may exist a similar regulation pathway in AML and CML which have been discovered in HCC. It had been demonstrated that miR-29b could impact CML cell proliferation and induces apoptosis via regulation of BCR/ABL1 protein and RNase-L [36,37]. Our data may provide more information about the mechanism of the effect of downregulating miR-29a/miR-29b in CML. Suggesting that Mcl-1 and Bcl-2 also regulated by miR-29a and miR-29b at post-transcriptional level and both of them contribute to the blocked apoptosis of leukemia cells in CML. Further research will focus on whether Mcl-1 and Bcl-2 could be directly target by miR-29 and miR-29b and how will they work in leukemia cells.

In conclusion, we characterized the expression feature of miR-29a/miR-29b and their target genes Mcl-1 and Bcl-2 in Chinese AML and CML patients. We also revealed that miR-29a/miR-29b negatively correlated with Mcl-1 and Bcl-2 in AML and CML. The data may support the finding of miR-29a and miR-29b as tumor suppressors. Further investigation is needed to confirm whether Mcl-1 and Bcl-2 could be directly regulate by miR-29, and whether miR-29 could be a effective biomarker and therapeutic target.

### Conclusions and future directions

Down-regulated miR-29a and miR-29b associated with up-regulated Bcl-2 and Mcl-1 in myeloid leukemias indicate that miR-29a/miR-29b may contribute to the anti-apoptosis of myeloid leukemia cells according to binding with 3’-UTR of Bcl-2 and Mcl-1 genes, further investigations will focus on the function of miR-29a and miR-29b in myeloid leukemia and their interaction with Bcl-2 and Mcl-1. Our current data support the role for miR-29a/29b dysregulation in myeloid leukemogenesis and the therapeutic promise of regulating miR-29a/29b expression for myeloid leukemia in the future.

## Abbreviations

AML: Acute myeloid leukemia; CML: Chronic myeloid leukemia; HI: Healthy individual Mcl-1, Myeloid cell leukemia sequence 1; Bcl-2: B-cell lymphoma 2; RT-PCR: Reverse transcription–polymerase chain reaction; PBMCs: Peripheral blood mononuclear cells; β2-MG: β2-microglobulin; LSCs: Leukemia stem cells; CMP: Common myeloid progenitors; CLL: Chronic lymphocytic leukemia; MCL: Mantle cell lymphoma; HCC: Hepatocellular carcinomas.

## Competing interests

The authors declare that they have no competing interests.

## Authors’ contribution

YQL directed and conceived the study and discussion. LX and YQL were involved in manuscript preparation. YX, ZYJ XW, XFZ, CWZ, SHC, LJY, GXL, BL performed a part of experiments. All authors reviewed and assisted in revising the manuscript. All authors read and approved the final manuscript.
